# Enhancing Antitumor Efficacy by Simultaneous ATP‐Responsive Chemodrug Release and Cancer Cell Sensitization Based on a Smart Nanoagent

**DOI:** 10.1002/advs.201801201

**Published:** 2018-10-26

**Authors:** Xiao‐Rong Song, Shi‐Hua Li, Hanhan Guo, Wenwu You, Datao Tu, Juan Li, Chun‐Hua Lu, Huang‐Hao Yang, Xueyuan Chen

**Affiliations:** ^1^ CAS Key Laboratory of Design and Assembly of Functional Nanostructures Fujian Key Laboratory of Nanomaterials Fujian Institute of Research on the Structure of Matter Chinese Academy of Sciences Fuzhou Fujian 350002 China; ^2^ MOE Key Laboratory for Analytical Science of Food Safety and Biology State Key Laboratory of Photocatalysis on Energy and Environment College of Chemistry Fuzhou University Fuzhou Fujian 350116 China

**Keywords:** antitumor, cell sensitization, chemodrugs, nanoagents, quantum dots

## Abstract

The exploitation of smart nanoagents based drug delivery systems (DDSs) has proven to be a promising strategy for fighting cancers. Hitherto, such nanoagents still face challenges associated with their complicated synthesis, insufficient drug release in tumors, and low cancer cell chemosensitivity. Here, the engineering of an adenosine triphosphate (ATP)‐activatable nanoagent is demonstrated based on self‐assembled quantum dots‐phenolic nanoclusters to circumvent such challenges. The smart nanoagent constructed through a one‐step assembly not only has high drug loading and low cytotoxicity to normal cells, but also enables ATP‐activated disassembly and controlled drug delivery in cancer cells. Particularly, the nanoagent can induce cell ATP depletion and increase cell chemosensitivity for significantly enhanced cancer chemotherapy. Systematic in vitro and in vivo studies further reveal the capabilities of the nanoagent for intracellular ATP imaging, high tumor accumulation, and eventual body clearance. As a result, the presented multifunctional smart nanoagent shows enhanced antitumor efficacy by simultaneous ATP‐responsive chemodrug release and cancer cell sensitization. These findings offer new insights toward the design of smart nanoagents for improved cancer therapeutics.

## 1 Introduction

Nanomaterials have gained numerous anticipated achievements in biomedicine, including recent surges in developing nanoagents based drug delivery system (DDS) to circumvent the nonspecific biodistribution of free drugs.^1^ However, there are some as yet unsolved problems that impede the therapeutic effect of the DDS, mainly including the insufficient accumulation of nanoagents and drugs in targeted sites as well as the unwanted side effects.^2^ The targeted delivery of nanoagents to tumor site is the primary desire. Although active targeting provides a promising way for drug nanocarriers to target tumors, the heterogeneous expression of specific receptors on tumor cells and the diversity of vessel structures in tumors limit the broad applicability of active targeting strategy.^3^ Besides, tumor‐specific drug release of DDS is desired to reduce the toxicity effect of drugs to normal tissues.^4^ Therefore, it is important but elusive to design more versatile and effective DDS with tumor targeting and reduced side effects.

In recent years, smart nanoagents based DDS in response to specific stimulus in tumors have attracted increasing interests.[[qv: 4a,5]] The therapy strategy is generally designed based on the hallmarks of tumor microenvironment (TM), such as hypoxia and dysregulated redox potential and enzymes.^6^ Given the high commonality of TM, smart nanoagents display distinct merits of broad applicability, enhanced therapeutic efficacy, and reduced side effects.^7^ In line with efforts to respond to the certain stimulus in TM, it is extremely complex and challenging to impart nanoagents with changeable structures through particular surface functionalization.^8^ As such, smart nanoagents constructed through a facile approach are actively being pursued.^9^

**Scheme 1:**
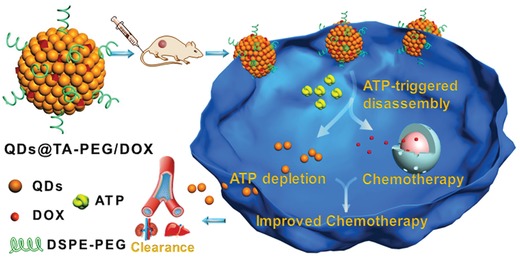
Schematic illustration of QDs@TA‐PEG/DOX for enhanced chemotherapy by simultaneous ATP‐responsive chemodrug release and cancer cell sensitization. The nanoagent was constructed via one‐step assembly of tannic acid, QDs, and DOX mediated by metal–phenolic coordination, which displayed ATP‐activated disassembly due to the strong metal–triphosphate coordination. Upon entering cancer cells, the nanoagent enabled ATP‐responsive drug release and cellular ATP depletion, resulting in the increased cell chemosensitivity and enhanced therapeutic efficacy without causing side effects to normal cells.

Apart from the effective delivery of drugs into cancer cells, biochemical modulation of cellular metabolism contributes to enhancing therapeutic efficacy.^10^ In this context, the modulation of intracellular adenosine triphosphate (ATP) level is of great interest. As an important molecule that provides energy supply for cell survival, ATP is highly concentrated in tumor cells (1–10 × 10^−3^ m) due mainly to the excessive glycolysis.^11^ ATP directly participates in many physiological processes, such as ATP‐driven proton pumps and ATP‐dependent drug efflux.^12^ Depleting intracellular ATP contents was found to effectively increase cancer cell chemosensitivity and enhance therapeutic efficacy of chemodrugs; however, it is challenging to design a multifunctional DDS with smart drug delivery and intracellular ATP depletion capabilities for improved chemotherapy.^13^ Herein, we for the first time engineer the self‐assembled quantum dots (QDs)‐phenolic nanoclusters (NCs) as an smart nanoagent for enhancing anti‐tumor efficacy by synergetic ATP‐responsive chemodrug release and cancer cell sensitization (**Scheme** **1**). Through the assembly of QDs, tannic acid (TA) and chemodrug mediated by the metal‐phenolic coordination,^14^ the developed nanoagent enables high chemodrug loading, ATP‐activated chemodrug release, high tumor accumulation, and body clearance. Significantly, we reveal a strategy for increasing cell chemosensitivity through cell ATP depletion induced by the nanoagent. As a result, efficient inhibition of tumor growth is realized in tumor‐bearing mice without causing evident side effects.

## 2 Results and Discussion

**Figure 1:**
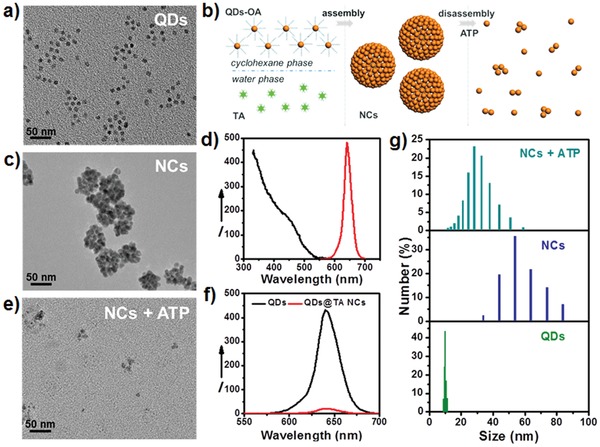
a) TEM image of hydrophobic CdSe@ZnS QDs. b) Schematic illustration of the assembly of QDs@TA NCs and ATP‐activated disassembly. c) TEM image of QDs@TA NCs. d) Excitation spectrum (black lines, λ_em_: 640 nm) and emission spectrum (red line, λ_ex_: 365 nm) of CdSe@ZnS QDs in cyclohexane. The “I” in *Y*‐axis was short for “intensity”. e) TEM image of the NCs after 20 × 10^−3^ m ATP treatment for 4 h. f) Emission spectra of QDs (black line) and QDs@TA NCs (red line) excited by 365 nm. g) DLS measurement of the related samples: QDs (bottom), QDs@TA NCs (middle), and QDs@TA NCs after 20 mM ATP treatment for 4 h (upper).

Hydrophobic core–shell structured CdSe@ZnS QDs with diameters of 9.82 ± 0.57 nm were synthesized on account of their enhanced chemical stability (**Figure** **1**a).^15^ Through blending cyclohexane solution of QDs with aqueous solution of TA under strong agitation, hydrophobic QDs were phase transferred into aqueous phase as schematically illustrated in Figure 1b. Characterizations through transmission electron microscopy (TEM), dynamic light scattering (DLS) and inductively coupled plasma atomic emission spectrometry (ICP‐AES) revealed the assembly of QDs into QDs@TA NCs with diameters of 55.1 ± 9.4 nm and a yield of about 80% (Figure 1c,g) due to that QDs can be linked by TA via the Zn‐phenolic coordination between ZnS shell and multiple phenolic groups in TA.[[qv: 14c]] Powder X‐ray diffraction patterns indicated the preservation of crystallinity after forming NCs (Figure S1, Supporting Information). Fluorescence analysis showed that hydrophobic QDs emitted a fluorescence peak centered at 640 nm with absolute quantum yield of 67.1% (Figure 1d). In contrast, the NCs in aqueous solution showed ≈90% quenching of fluorescence compared to that of QDs in cyclohexane solution due to the energy transfer between the assembled QDs within short distance and fluorescence quenching effect of catechol groups in TA (Figure 1f).^16^ Because of the free phenolic groups in surface TA, QDs@TA NCs carried a zeta potential of −28 mV and displayed good water dispersibility (Figure S2, Supporting Information).

**Figure 2:**
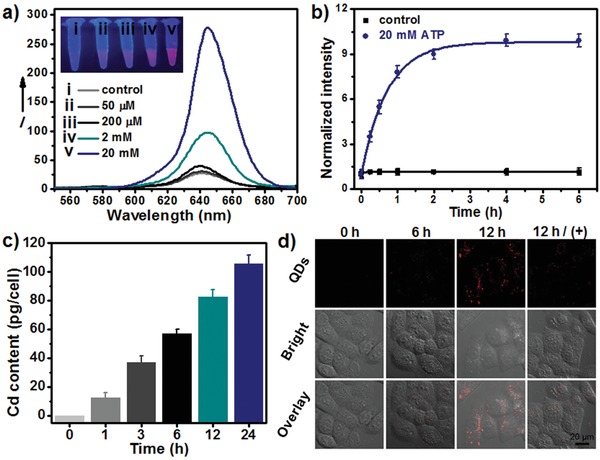
a) Emission spectra of QDs@TA NCs treated with different concentrations of ATP. Inset: images of various samples under 365 nm light irradiation. b) Time evolution of the fluorescence intensity of QDs@TA NCs in PBS (control) and 20 × 10^−3^ m ATP, respectively. c) Time evolution of cellular uptake after incubating the HepG2 cells with QDs@TA‐PEG (100 µg mL^−1^) for various time periods. d) Confocal images of HepG2 cells after treating with QDs@TA‐PEG for different time periods (0, 6, and 12 h). (+): with apyrase treatment. Images were acquired using an exication laser of 408 nm and emission range from 570–1000 nm.

Due to the strong binding of ATP to various metal ions through metal ion‐triphosphate coordination, it is anticipated that ATP may compete with TA and partly displace TA to bind on QDs surface. Upon ATP treatment, we discovered that the intact NCs were disassembled into single QDs or small clusters (Figure 1e,g). The ATP‐activated disassembly of the NCs was reasonable due to the fact that the binding of ATP to metal sulfide QDs through metal–triphosphate coordination was stable enough to passivate QDs with good water dispersibility, thus resulting in the disassembly of QDs by replacing TA with ATP on QDs surface.^17^ The disassembled NCs displayed intensified fluorescence as the increase of treated ATP concentration because of the inhibition of energy transfer between disassembled QDs and the separation of TA from QDs surface (**Figure** **2**a). Through kinetic study of fluorescence recovery, it was observed that the fluorescence intensity was gradually enhanced and reached a plateau at 4 h of incubation with 20 × 10^−3^ m ATP (Figure 2b). Particularly, the recovered fluorescence showed linear correlation with ATP concentration ranging from 50 × 10^−6^ m to 5 × 10^−3^ m (Figure S3, Supporting Information). Further control experiments revealed the high selectivity of turn‐on fluorescence for ATP over adenosine monophosphate (AMP) and adenosine diphosphate (ADP) (Figure S4, Supporting Information). We also found that uridine triphosphate (UTP), cytidine triphosphate (CTP), and guanosine triphosphate (GTP) could trigger comparable enhancement of fluorescence relative to ATP (Figure S4, Supporting Information), suggesting that the disassembly of NCs was stimulated by the metal ion–triphosphate coordination. It is worth noting that the intracellular levels of these ATP analogues are approximately threefold to tenfold lower than that of ATP.^18^ Thus, such ATP‐activated disassembly with turn‐on fluorescence showed great potential applications in tumor‐specific drug delivery and fluorescence imaging due to the highly elevated ATP contents in cancer cells.^19^

To improve the biocompatibility of QDs@TA NCs, phospholipid‐polyethylene glycol (PEG) was further functionalized on the NCs mediated by the hydrophobic interaction between the phospholipid terminal and multiple phenyl groups in TA.^20^ The PEG modified NCs (QDs@TA‐PEG) exhibited a relatively weaker charge value of about −16 mV than that of the NCs because of the charge screening effect of surface PEG (Figure S2, Supporting Information). To survey the ATP‐responsive disassembly of the NCs after PEG modification, we carried out fluorescence imaging and DLS measurement. Fluorescence images indicated the ATP‐activated turn‐on fluorescence of QDs@TA‐PEG (Figure S5a, Supporting Information). DLS data revealed the size decrease of the QDs@TA‐PEG after ATP treatment, indicating that PEG modified NCs can be disassembled by ATP (Figure S5b, Supporting Information). DLS data also revealed that QDs@TA‐PEG maintained high stability in water, phosphate buffered saline (PBS), and cell culture media without apparent size change even after 10 days' storage (Figures S5c and S6, Supporting Information). Furthermore, cellular uptake and cytotoxicity of QDs@TA‐PEG NCs were surveyed on hepatocellular carcinoma cell lines (HepG2). Cellular uptake of the NCs were elevated with the increase of incubation time and reached about 105.6 ± 6.2 pg per cell of Cd content at time point of 24 h (Figure 2c). Through cytotoxicity test, no evident cytotoxic effect was shown on HepG2 cell viability (>90%) even when the concentration of QDs@TA‐PEG NCs reached 120 µg mL^−1^ (Figure S7, Supporting Information). Such low cytotoxicity of the NCs can be ascribed to the chemical stability of QDs and the inhibited release of toxic Cd ions (<1%) (Figure S8, Supporting Information). It should be pointed out that although TA itself can kill cancer cells at dosages exceeding 50 µg mL^−1^ (Figure S9, Supporting Information),^21^ the low mass content of TA in QDs@TA‐PEG (≈10.3%) ensured the low cytotoxicity of QDs@TA‐PEG (Figure S10, Supporting Information). These studies demonstrated the high cellular uptake of QDs@TA‐PEG without causing cytotoxicity.

In virtue of ATP‐activated turn‐on fluorescence, QDs@TA‐PEG NCs were then served as fluorescent probes for ATP imaging in living cells. Confocal fluorescence images showed the increase of cellular fluorescence with the prolongation of time after the incubation of QDs@TA‐PEG (Figure 2d). To verify the potential of the NCs for monitoring intracellular ATP level, HepG2 cells were cultured with the NCs in the presence of apyrase, which can induce the reduction of intracellular ATP contents by catalyzing the hydrolysis of ATP.^22^ Cellular uptake study indicated that cellular NCs contents were not obviously affected with apyrase treatment (Figure S11, Supporting Information). Significantly, cellular fluorescence was diminished in cells treated with apyrase compared to that without apyrase treatment (Figure 2d), suggesting the specific imaging of intracellular ATP level.

**Figure 3:**
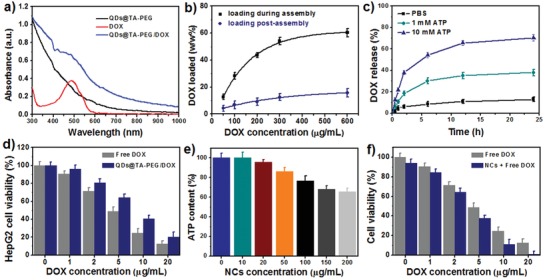
a) UV–vis–NIR absorbance spectra of QDs@TA‐PEG, DOX and QDs@TA‐PEG/DOX. b) The DOX‐loading capability of QDs@TA‐PEG NCs under various DOX feeding amounts and different loading manner. c) Time evolution of DOX release profiles of QDs@TA‐PEG/DOX under different conditions. d) Relative HepG2 cell viability after treating with QDs@TA‐PEG/DOX or free DOX for 24 h. e) ATP contents in HepG2 cells cultured with different concentrations of the NCs for 24 h. f) HepG2 cell viability after the treatment of different dosages of DOX plus 100 µg mL^−1^ NCs for 24 h.

Drug loading and release behaviors of QDs@TA‐PEG were subsequently examined by choosing doxorubicin (DOX) as a model drug. After blending DOX, QDs, and TA in the two‐phase solution, we obtained DOX‐loaded NCs (QDs@TA‐PEG/DOX). Absorption spectrum of QDs@TA‐PEG/DOX displayed a characteristic absorption of free DOX around 480 nm (**Figure** **3**a). The DOX loading capacity of the NCs was heightened with the increase of DOX feeding amounts and reached about 60% (w/w%) (Figure 3b). Zeta potential measurements indicated a decrease of the negative potential of QDs@TA‐PEG/DOX as the increase of DOX loading amounts because of the positive potential of DOX (Figure S12, Supporting Information). We then investigated the stability of loaded DOX by dispersing QDs@TA‐PEG/DOX in PBS and cell culture media. The data showed only ≈16% release of DOX after 1 week's incubation, indicating the good stability of loaded DOX under biological conditions (Figure S13a, Supporting Information). A different strategy was next employed for loading DOX in the NCs through mixing DOX with the already formed NCs, resulting in the DOX‐loading capacity of only 14.6% (Figure 3b), much lower than that loaded during the assembly process of the NCs. It can thus be inferred that DOX was mainly located in the interior regions of the NCs after blending DOX, QDs, and TA in two‐phase solutions. Significantly, upon treating QDs@TA‐PEG/DOX with ATP, released DOX amounts were gradually increased as the increase of ATP concentration and the prolongation of incubation time (Figure 3c). Specifically, we achieved 70% release of DOX with the treatment of 10 × 10^−3^ m ATP for 24 h, revealing the ATP‐activated drug release and the potential for ATP‐activated chemotherapy.^23^ Under same conditions, AMP and ADP induced slight release of DOX (<18%), while UTP, CTP, and GTP resulted in comparable release of DOX as ATP (Figure S13b, Supporting Information), indicating the triphosphate‐specific drug release.

The chemotherapeutic effect of QDs@TA‐PEG/DOX was then evaluated on cancer cells and normal cells. Cell counting kit‐8 (CCK‐8) results revealed the dosage‐dependent chemotherapeutic efficacy of both free DOX and QDs@TA‐PEG/DOX (Figure 3d). QDs@TA‐PEG/DOX had weaker therapeutic efficacy than that of free DOX at same dosage due to the incomplete DOX release in QDs@TA‐PEG/DOX. By taking hepatocellular lines (L02) as normal cells, the 50% inhibiting concentration (IC_50_) of free DOX to L02 cells was calculated to be 3.96 µg mL^−1^, lower than that to HepG2 cells (4.61 µg mL^−1^), implying that normal cells exhibited higher chemosensitivity than cancer cells. Interestingly, L02 cells had cell viability of 63% with the treatment of QDs@TA‐PEG/DOX containing 20 µg mL^−1^ DOX (Figure S14, Supporting Information), which can be owing to the low cellular uptake of QDs@TA‐PEG/DOX in L02 cells (Figure S15, Supporting Information) and the inhibited DOX release under low ATP contents. We further incubated L02 cells with ATP after the uptake of QDs@TA‐PEG/DOX. Cytotoxicity test showed the reduced cell viability with the increase of added ATP concentration, which suggested that additional ATP supply in L02 cells can effectively induce cell death. Thus, low cytotoxicity of QDs@TA‐PEG/DOX can be attributed to the low ATP contents in L02 cells (Figure S16, Supporting Information). In this sense, QDs@TA‐PEG/DOX was capable to kill cancer cells without causing side effects to normal cells.

To reveal the therapeutic action of QDs@TA‐PEG/DOX, cancer cells were subjected to lactate dehydrogenase (LDH) leakage assay and ATP content measurement after the treatment of QDs@TA‐PEG NCs. LDH level in cells with NCs treatment was not obviously changed compared to that in control cells, indicating that the NCs did not impact the integrity of cell membrane (Figure S17, Supporting Information). Through ATP content measurement of cells treated with the NCs for 24 h, we found a decrease of intracellular ATP content with the increase of NCs concentration. Particularly, intracellular ATP contents showed a depletion of 24% after treating HepG2 cells with 100 µg mL^−1^ NCs for 24 h (Figure 3e). Considering the potential of ATP depletion for enhancing cell chemosensitivity and anticancer efficacy of chemodrugs,^13^ we then examined HepG2 cell viability after treating the cells with free DOX plus QDs@TA‐PEG NCs. CCK‐8 results showed that free DOX plus QDs@TA‐PEG treatments resulted in not only higher therapeutic efficacy than that of free DOX to HepG2 cells, but also a decrease of HepG2 cell viability with the increase in NCs concentration, validating enhanced cell chemosensitivity with the treatment of QDs@TA‐PEG (Figure 3f and Figure S18, Supporting Information). Therefore, the therapeutic action of QDs@TA‐PEG/DOX can be attributed to the synergetic effect of intrinsic cytotoxicity of DOX and ATP depletion enhanced chemosensitivity of cancer cells. The design of smart drug nanocarriers functionalized with unique ATP depletion will provide an effective strategy for improving chemotherapy.

**Figure 4:**
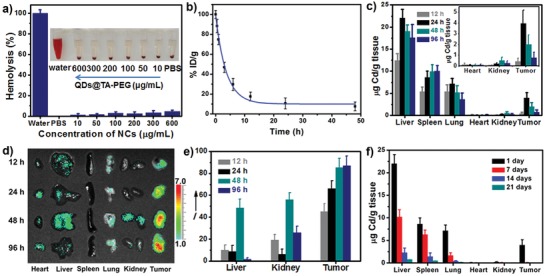
a) Hemolysis assay of RBCs treated with water, PBS, and different concentrations of QDs@TA‐PEG NCs for 3 h. Inset: Photographs of corresponding solutions after centrifugation. b) Blood circulation time study of QDs@TA‐PEG in tumo‐bearing mice. c) Biodistribution of the NCs in major organs and tumors after IV injection of QDs@TA‐PEG for 12, 24, 48, 96 h. d) Fluorescence images of major organs and tumors collected at 12, 24, 48, and 96 h post IV injection. The fluorescent bar was shown in false colour scale (1 unit = 1 × 10^6^ photons per s per cm^2^ per sr). e) Fluorescence intensity of the corresponding samples presented in panel (d) quantitated by Image J software. f) Biodistribution of the NCs in major organs and tumors at 1, 7, 14, and 21 d post IV injection.

The blood circulation time and in vivo tissue biodistribution of QDs@TA‐PEG NCs were next studied before in vivo antitumor study. Hemolysis test revealed no overt hemolysis in mice red blood cells (RBCs) after NCs treatments, which suggested the biocompatibility of QDs@TA‐PEG (**Figure** **4**a). NCs contents in blood and tissue samples were determined by ICP‐AES after the digestion of blood and tissue samples. The blood circulation time of QDs@TA‐PEG NCs was measured to be about 2.9 h (Figure 4b). Through subsequent biodistribution study, we found an increase of NCs contents in liver, spleen, lung, and tumors from 12 to 24 h post intravenous (IV) injection, and a decrease of NCs contents in such tissues from 24 h to 96 h post IV injection (Figure 4c). It was observed that kidney tissue displayed 5‐fold and 2.5‐fold increase of NCs contents at 48 h compared to that at 12 and 24 h, respectively. The fluorescence in major organs and tumors was then investigated by fluorescence imaging. The data revealed that tumor displayed 2.8‐fold enhancement of fluorescence at 48 h compared to that at 12 h post IV injection (Figure 4d,e). In view of the decrease of the NCs contents in tumor after 24 h post IV injection, such enhancement of fluorescence in tumor was very likely caused by the disassembly of QDs@TA‐PEG. We also found an obvious fluorescence in kidney at 48 h post IV injection, which revealed the renal clearance of the disassembled QDs. Long‐term in vivo biodistribution study showed a gradual decrease of NCs contents in all major organs and tumors in three weeks, revealing the excretion of the NCs from body (Figure 4f). These results demonstrated that QDs@TA‐PEG NCs were able to be highly accumulated in tumors and finally cleared out from body without potential long‐term toxicity.

**Figure 5:**
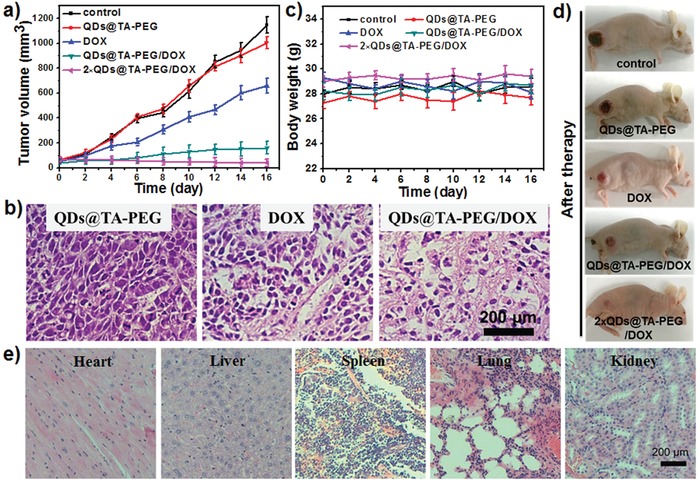
a) Time evolution of tumor volume in mice of different groups: PBS (control), QDs@TA‐PEG, DOX, QDs@TA‐PEG/DOX and 2 × QDs@TA‐PEG/DOX, respectively (DOX: 5 mg kg^−1^; NCs: 20 mg kg^−1^). b) H&E stained histological images of tumors collected at day 2. c) Body weights of mice during various treatments. d) Representative photos of mice after 16 days' treatment. e) H&E stained histological images of major organs collected at day 16 in mice of QDs@TA‐PEG/DOX group.

Finally, tumor‐bearing mice were divided into five groups for antitumor chemotherapy. When the tumor size reached about 60 mm^3^, mice were intravenously injected with various formulations. It was observed that tumors in control group, DOX group and single NCs group grew rapidly with about 18, 16, and 10‐fold increase of tumor volume after 16 days' treatment, respectively (**Figure** **5**a,d). In sharp contrast, we found only a 2.5‐fold increase of tumor volume in mice with single injection of QDs@TA‐PEG/DOX, suggesting its better inhibition of tumor growth than that of other three groups. Significantly, the growth of tumors can be completely inhibited when performing tumor therapy with twice injection of QDs@TA‐PEG/DOX (first injection: day 0, second injection: day 4; Figure 5a). Hematoxylin and eosin (H&E) staining revealed that obvious cell deformation and shrinking nucleus were observed in tumors after 2 days' treatment of QDs@TA‐PEG/DOX, while single QDs@TA‐PEG or DOX cannot sufficiently induce cell damage (Figure 5b). In all treatments, mice had normal weights without weight loss, which further implied the reduced side effect of QDs@TA‐PEG/DOX (Figure 5c). Besides, QDs@TA‐PEG/DOX was gradually excreted out from body in 16 days' treatment, which was coincident with the results from the counterparts without DOX loading (Figure S19, Supporting Information). Furthermore, histology analysis revealed that no overt damage or inflammatory lesion in major organs occurred after 16 days' treatments in all groups (Figure 5e and Figure S20, Supporting Information). These findings verified the complete inhibition of tumor growth and unapparent side effects of QDs@TA‐PEG/DOX.

## 3 Conclusion

In brief, we have rationally designed a unique smart nanoagent based on QDs@TA‐PEG/DOX for improved antitumor chemotherapy through combining cancer‐specific controlled drug delivery and cancer cell sensitization. The nanoagent is constructed through a one‐step approach mediated by the metal–phenolic coordination and features high drug loading capacity. The developed nanoagent can be distinctly disassembled by ATP activation, thus enabling ATP‐activated controlled drug release in cancer cells with reduced toxicity to normal cells. More strikingly, the nanoagent can deplete the intracellular ATP content and increase cell chemosensitivity for raising therapeutic efficacy. With high tumor accumulation and body clearance of the nanoagent, we successfully realize the complete inhibition of tumor growth without evident side effects. This study presents the design of smart nanoagents with improved cancer chemotherapy, which may accelerate the exploitation and clinical translation of smart therapeutic nanoagents.

## 4 Experimental Section

*Chemicals*: ATP (99%), ADP (95%), AMP (97%), TA, apyrase, cadmium oxide (CdO, 99.5%), selenium power (99.99%), sulfur powder (99.9%), zinc acetate (ZnAc_2_, 99.99%), trioctylphosphine (TOP, 90%), oleic acid (OA, 90%), and 1‐octadecene (ODE, 90%) were purchased from Sigma‐Aldrich. CCK‐8 was purchased from Beyotime Institute of Biotechnology (China). Distearoyl phosphatidylethamolamine‐*N*‐poly(ethyleneglycol) 2000 (DSPE‐PEG, Mw 2000) was purchased from Xi'an Ruixi Biological Technology Co. Ltd (China). Other chemicals were of analytical grade and used as purchased. Ultrapure water obtained from a Millipore water purification system was used in all runs.

*Synthesis of CdSe/ZnS Core–Shell QDs*: In the synthesis, Cd(OA)_2_ and Zn(OA)_2_ were obtained as precursors by mixing CdO (0.4 mmol), ZnAc_2_ (4 mmol), and OA (17.6 mmol) with ODE (20 mL), followed by degassing, N_2_ gas filling, and heating to 310 °C. TOP solution (3 mL) containing Se (0.4 mmol) and S (4 mmol) was then quickly injected into the above solution of precursors for growth. The CdSe@ZnS QDs were obtained after reaction for 30 min, which was further purified by washing with chloroform and acetone for three times.

*Preparation of QDs@TA NCs*: Aqueous solution of TA was mixed with an equal volume of cyclohexane solution of QDs, followed by stirring and sonication for 4 h. After stratification, the resulting aqueous phase solution was then collected. After centrifugation and washing for three times with ethanol and water, QDs@TA NCs were obtained and stored at 4 °C for further use. For DSPE‐PEG modification, DSPE‐PEG (Mw 2000, 5 mg mL^−1^) was stirred with the NCs overnight, followed by centrifugation and washing to obtain QDs@TA‐PEG NCs.

*Drug Loading and Release in QDs@TA‐PEG NCs*: To load drugs during assembly process, aqueous solution of TA and DOX was mixed with cyclohexane solution of QDs, followed by stirring and sonication for 4 h. By loading drugs after the formation of the NCs, QDs@TA NCs were stirred with aqueous solutions of DOX for 4 h to obtain QDs@TA‐PEG/DOX. The DOX loading amount was quantified by subtracting DOX mass in supernatant from total DOX mass. The loading capacity was expressed by mass percentage relative to the mass of NCs. DSPE‐PEG modification was also carried out as mentioned above. For studying drug release behavior, aqueous solution of QDs@TA‐PEG/DOX (5 mL) in dialysis bag (*M_w_* 3000) was immersed into buffered solution (pH 7.0, 20 mL) containing different concentrations of ATP. At desired time points, dialysis solutions (1 mL) were collected. The DOX amount was calculated from its absorbance and used for determining DOX loading and release capability.

*In Vitro Cell Experiments*: Cells were cultured in RPMI‐1640 medium (Gibco) with 10% fetal bovine serum (Gibco) at 37 °C in a humidified atmosphere with 5% CO_2_. For cytotoxicity test of NCs, cells were seeded in 96‐well plates with 10^4^ cells per well. In a typical cytotoxicity test of QDs@TA‐PEG, HepG2 cells were incubated with culture media containing different concentrations of QDs@TA‐PEG for 24 h. After replacing the culture media with fresh media containing CCK‐8 solution, cells were further cultured for 0.5 h and then subjected to absorbance measurement to determine the cell viability according to the manufacture's protocol. To compare the therapeutic effect of free DOX and DOX loaded NCs, QDs@TA‐PEG/DOX with about 25% DOX loading was used as a model. The 50% inhibitory concentrations (IC_50_ values) were calculated by Probit analysis method using the SPSS software. For cellular uptake study, HepG2 cells were seeded in sixwell plates with 10^5^ cells per well. Thereafter, HepG2 cells were cultured with QDs@TA‐PEG (100 µg mL^−1^) for different time periods, followed by washing with PBS. The collected cells were next treated with aqua regia and diluted to aqueous solutions (10 mL) for measuring Cd contents by ICP‐AES. For cell confocal imaging, HepG2 cells were seeded in 20 mm confocal dishes. After incubating cells with QDs@TA‐PEG (30 µg mL^−1^) for different time periods, cells were washed with PBS for three times and imaged by confocal laser scanning microscopy immediately.

*LDH Leakage and ATP Assay*: LDH leakage was examined using a LDH release assay kit (Beyotime Institute of Biotechnology) following the manufacture's protocol. In the experiment, HepG2 cells were incubated with different concentrations of the NCs for 24 h. The positive control was generated by incubating cells with 2% Triton X‐100 for 30 min. The negative control was generated by using the cell‐free medium. The LDH content was measured at 490 nm with a microplate reader. For determining intracellular ATP level, cells were seeded in 12‐well plates at a density of 2 × 10^5^ cells per well. Various concentrations of the NCs in cell culture media were added to cells and incubated for 24 h. Cells were then washed twice with PBS and lysed. ATP contents in cell lysates were then determined by using ATP assay kit (Beyotime Institute of Biotechnology).

*Hemolysis Test*: Hemolysis assay was carried out by using mice RBCs as model cells. RBCs were isolated and washed with PBS for five times. Then different concentrations of QDs@TA‐PEG were added into the cell suspensions of the obtained RBCs for 3 h. The released hemoglobin in the supernatant was determined through measuring its absorbance at 540 nm. The extent of hemolysis was calculated relative to 100% hemolysis treated with ultrapure water.

*In Vivo Experiments*: All experiments involving animals were implemented according to the relevant laws and institutional guidelines, and were approved by the Animal Ethics Committee of Fujian Medical University. For pharmacokinetic study, blood of mice was collected at different time points (0, 0.5, 1, 2, 6, 12, and 24 h) after IV injection of QDs@TA‐PEG at a dosage of 20 mg kg^−1^. The collected samples were digested and diluted for ICP‐AES test to calculate Cd contents. For biodistribution study, major organs and tumors of mice were collected at different time points post IV injection (dosage: 20 mg kg^−1^). The collected tissues were weighed, homogenized, and digested in a mixture solution of nitric acid and hydrogen peroxide solution (6:1, v/v) to release Cd ions. The contenst of the NCs in each tissue were determined by ICP‐AES and described as microgram of Cd content per gram of tissue (Cd µg per tissue g). For fluorescence imaging of tissues, major organs and tumors of mice were also collected at different time points postinjection, and then imaged by an SI Imaging Amix small animal imaging system (Spectral Instruments Imaging Co., USA). Fluorescence intensity was analyzed by the Image J software. For tumor therapy, when the tumor size reached about 60 mm^3^, mice were treated with IV injection of free DOX and QD@TA‐PEG/DOX respectively (DOX: 5 mg kg^−1^; QD@TA‐PEG: 20 mg kg^−1^). Tumor volumes were calculated through ab^2^/2, where a and b represent the maximum diameter and the minimum diameter of tumor, respectively. The body weights of mice were also measured every other day. Major organs and tumors were collected for histological examination by H&E staining.

## Conflict of Interest

The authors declare no conflict of interest.

## Supporting information

Supplementary
