# Supplementary material for: Enhancing Antitumor Efficacy by Simultaneous ATP‐Responsive Chemodrug Release and Cancer Cell Sensitization Based on a Smart Nanoagent
Source: Adv Sci (Weinh). 2018 Oct 26;5(12):1801201. doi: 10.1002/advs.201801201 (PMC6299707; doi:10.1002/advs.201801201)
Supplement: Supplementary file 1 — Supplementary [file ADVS-5-1801201-s001.pdf]

Copyright WILEY-VCH Verlag GmbH & Co. KGaA, 69469 Weinheim, Germany, 2018.

ADVANCED SCIENCE
Open Access

Supporting Information

for *Adv. Sci.*, DOI: 10.1002/advs.201801201

Enhancing Antitumor Efficacy by Simultaneous ATP-Responsive Chemodrug Release and Cancer Cell Sensitization Based on a Smart Nanoagent

Xiao-Rong Song, Shi-Hua Li, Hanhan Guo, Wenwu You, Datao Tu, Juan Li, Chun-Hua Lu, Huang-Hao Yang, and Xueyuan Chen**

Copyright WILEY-VCH Verlag GmbH & Co. KGaA, 69469 Weinheim, Germany, 2016.

Supporting Information

Enhancing Anti-Tumor Efficacy by Simultaneous ATP-Responsive Chemodrug Release and Cancer Cell Sensitization Based on A Smart Nanoagent

Xiao-Rong Song, Shi-Hua Li, Hanhan Guo, Wenwu You, Datao Tu, Juan Li, Chun-Hua Lu, Huang-Hao Yang, and Xueyuan Chen**

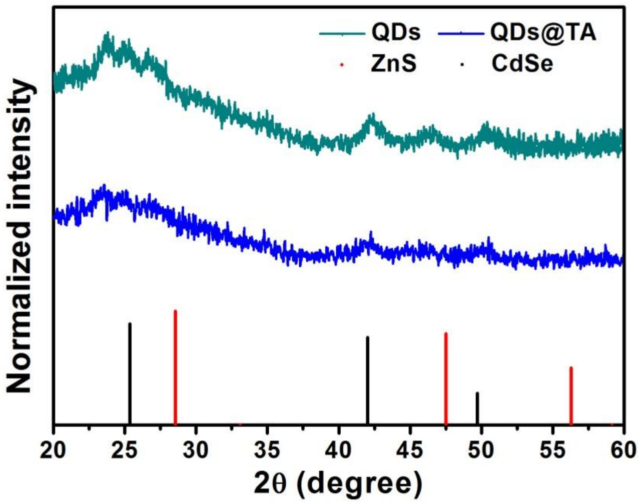

Figure S1. XRD patterns of as-synthesized CdSe@ZnS QDs and QDs@TA NCs.

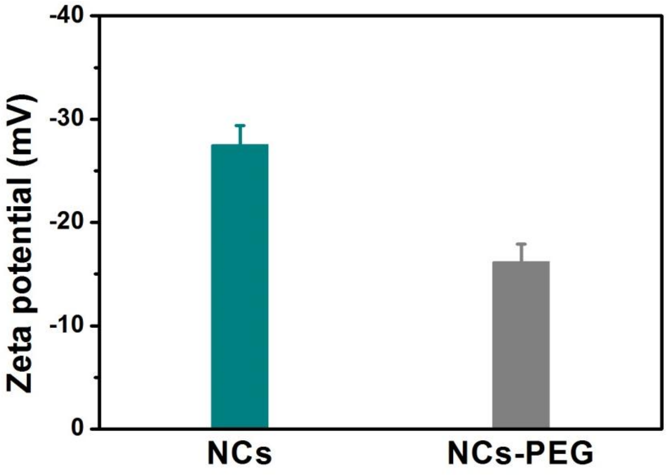

Figure S2. Zeta potentials of QDs@TA NCs and QDs@TA-PEG NCs.

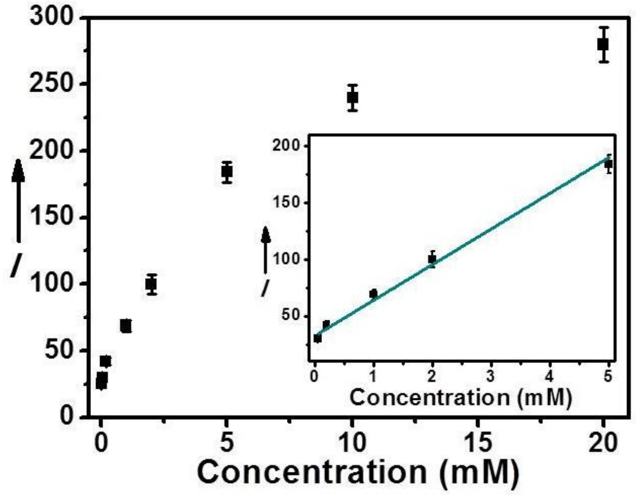

Figure S3. Fluorescence intensity of QDs@TA NCs treated with different concentrations of ATP ranging from 0 to 20 mM for 4 h.

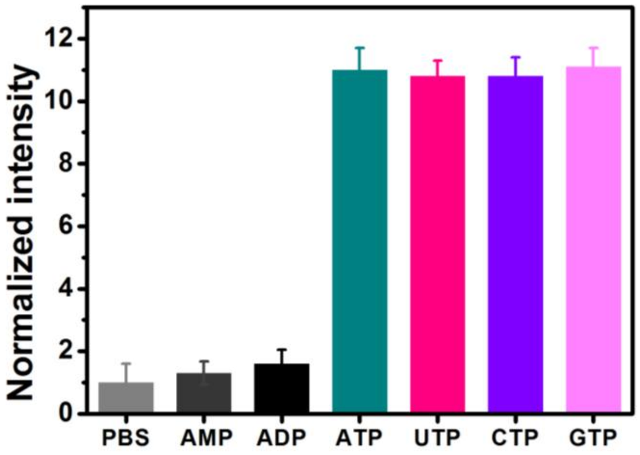

Figure S4. Normalized intensity of QDs@TA NCs after being treated with PBS, AMP, ADP, ATP, UTP, CTP, and GTP at concentration of 20 mM.

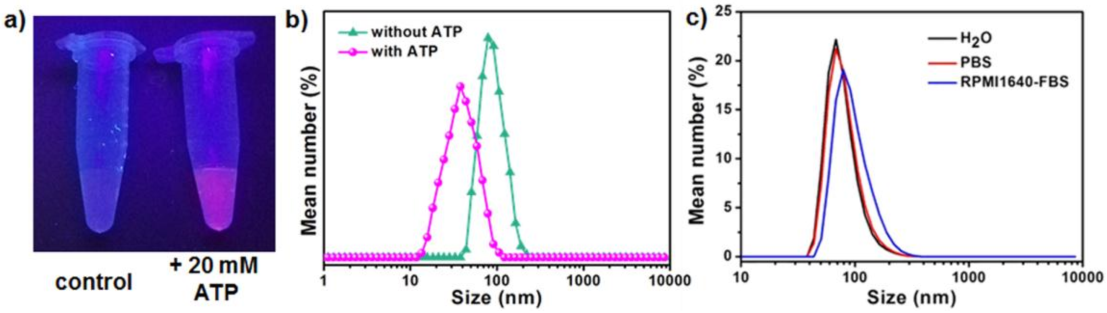

Figure S5. a) Images of QDs@TA-PEG solutions under 365 nm light irradiation without or with ATP treatments. b) DLS measurement of QDs@TA-PEG before and after ATP treatment. c) DLS measurement of QDs@TA-PEG dispersed in various solutions including water, PBS and cell culture media.

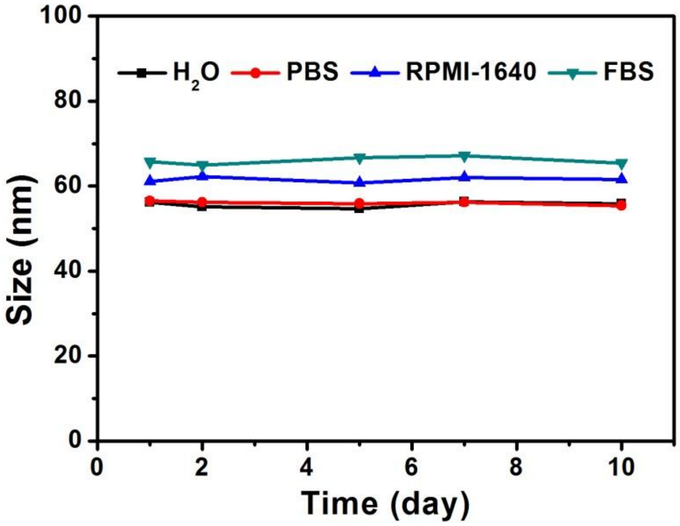

Figure S6. Time evolution of the size of QDs@TA-PEG after dispersing in various solutions as revealed from DLS measurement.

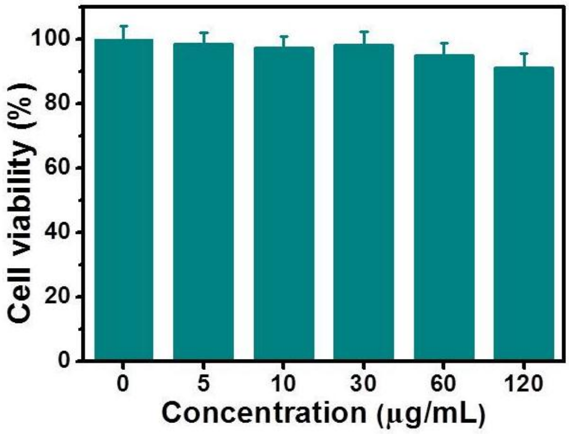

Figure S7. Cell viability of HepG2 cells after treating cells with various concentrations of QDs@TA-PEG for 24 h.

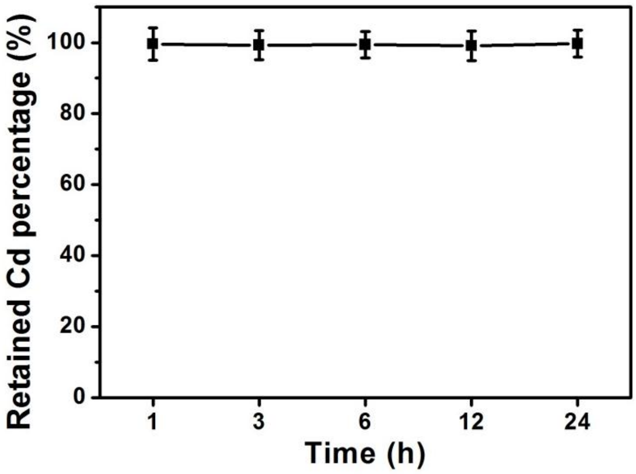

Figure S8. Retained Cd percentage in QDs@TA-PEG NCs after dispersing in cell culture media for different time periods. The measurement was carried out by incubating the QDs@TA-PEG NCs with cell culture media for different time periods, followed by centrifugation and washing. Cd contents in the various supernatants were determined by ICP-AES. Cd percentage was calculated as follow: (Cd contents in supernatants) / (total Cd contents in QDs@TA-PEG) ×100%.

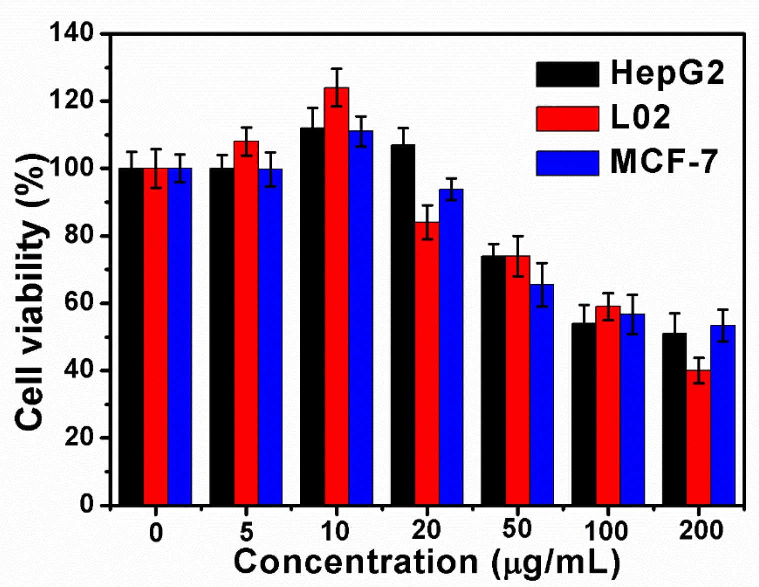

Figure S9. Relative cell viability of cancer cells after treating with various concentrations of TA for 24 h. The cell viability was determined by CCK-8.

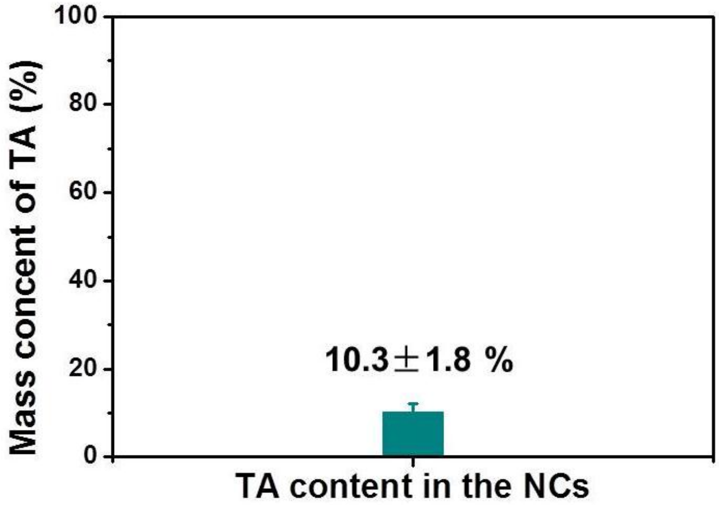

Figure S10. Mass content of TA in QDs@TA-PEG NCs determined by the Folin-Ciocalteu method. The calculation procedure was as follows: Firstly, standard solutions of TA with various concentrations were freshly prepared (0, 5, 10, 20, 50, 100, 200 μg/mL). After incubating the aqueous solutions of TA or QDs@TA-PEG NCs with Folin-Ciocalteu reagent and sodium carbonate solution for 1 h, the absorbance of the mixture at 725 nm was recorded respectively. The TA content in QDs@TA-PEG NCs was calculated from the obtained standard curve.

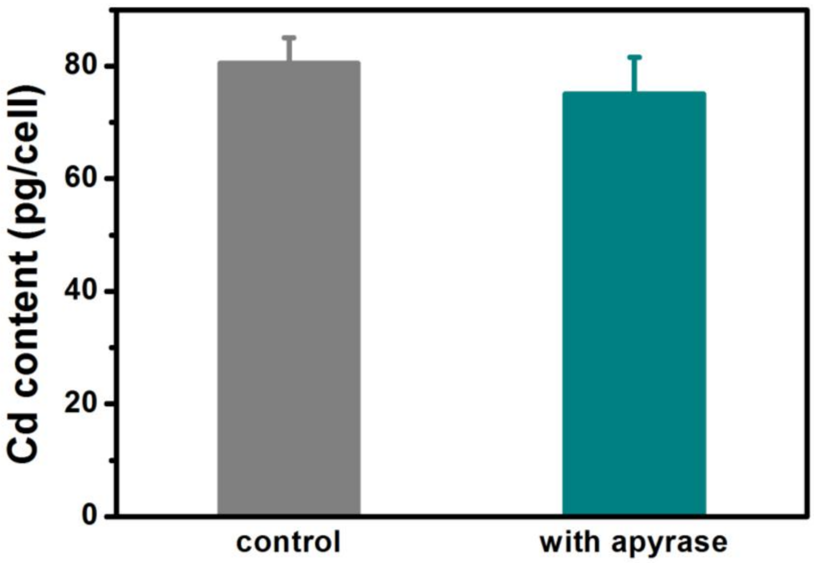

Figure S11. Intracellular uptake of the NCs after incubating HepG2 cells with the NCs for 12 h with or without apyrase treatment.

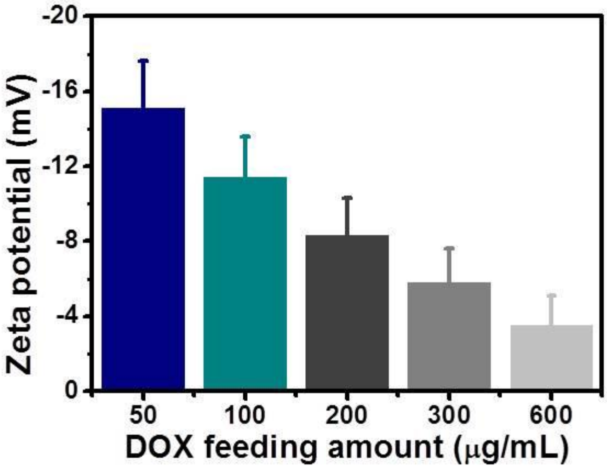

Figure S12. Zeta potentials of QDs@TA-PEG/DOX obtained under different DOX feeding amounts.

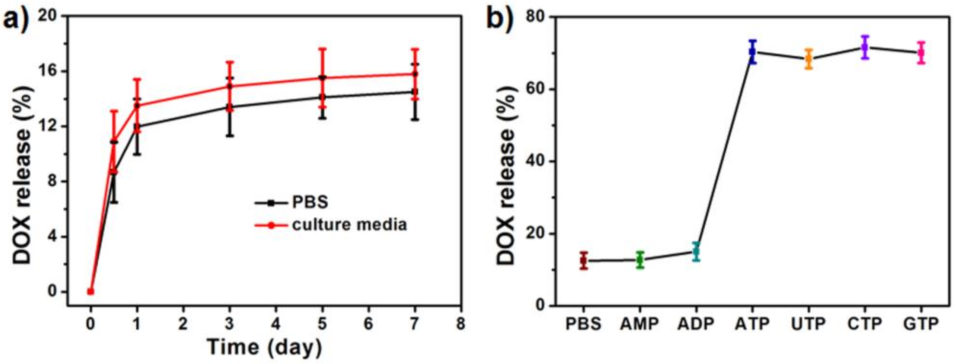

Figure S13. a) Time evolution of DOX release profiles of QDs@TA-PEG/DOX under PBS and cell culture media condition. b) DOX release of QDs@TA-PEG/DOX under PBS, AMP, ADP, ATP, UTP, CTP and GTP treatments, respectively (10 mM, 24 h).

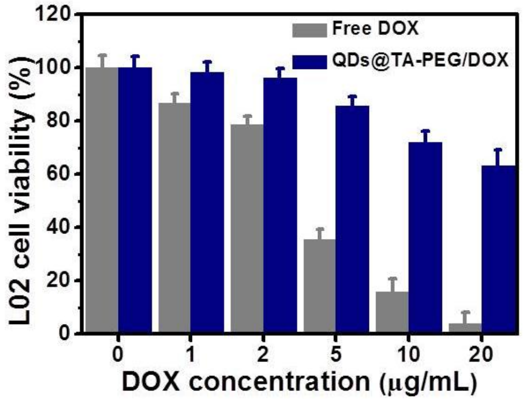

Figure S14. Relative viability of L02 cells after treating with free DOX or QDs@TA-PEG/DOX for 24 h.

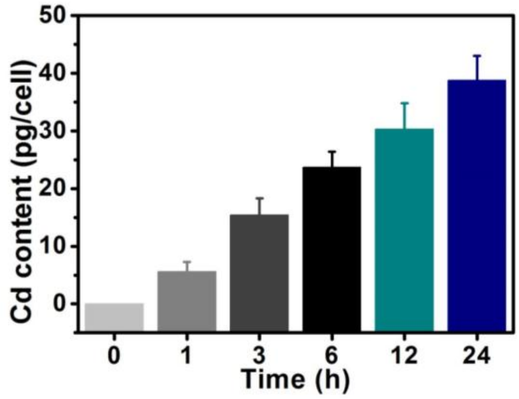

Figure S15. Time evolution of intracellular uptake after incubating the L02 cells with QD@TA-PEG for various time periods.

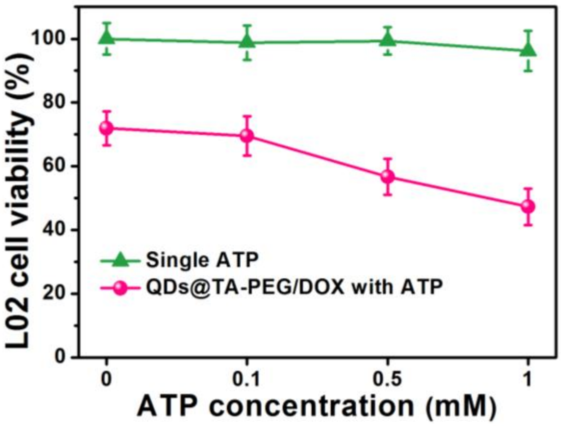

Figure S16. Cell viability of L02 cells after the uptake of QD@TA-PEG/DOX and further incubation with different concentrations of ATP (0.1, 0.5, and 1 mM).

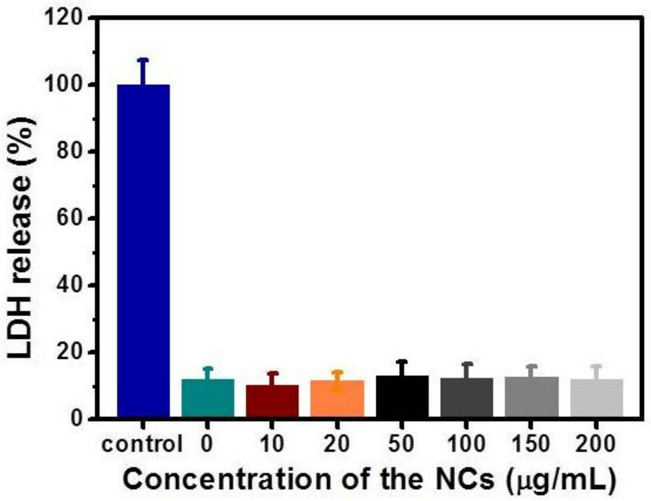

Figure S17. LDH leakage of HepG2 cells after culturing with different concentrations of the NCs for 24 h. The control with 100% LDH leakage was generated by treating cells with 2% Triton X-100 for 30 min.

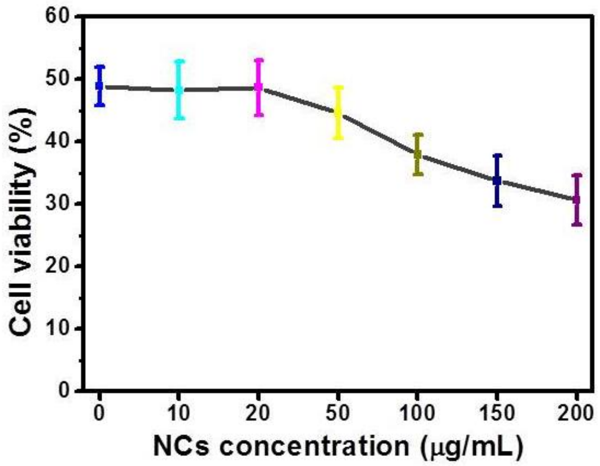

Figure S18. Cell viability of HepG2 cells after culturing with different concentrations of the NCs plus 5 μg/mL DOX for 24 h.

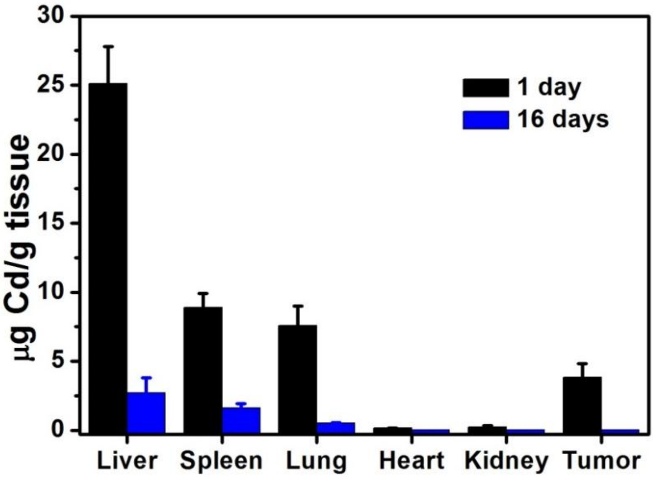

Figure S19. Biodistribution of Cd content in major organs and tumors after intravenous injection of QD@TA-PEG/DOX for 1 day and 16 days, respectively.

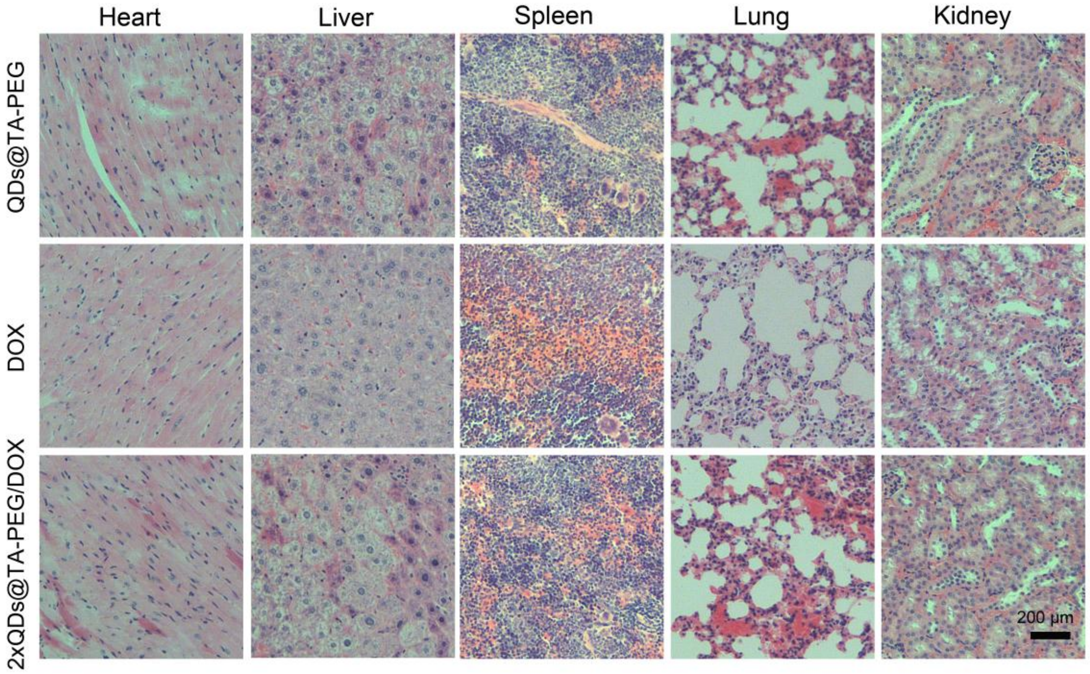

Figure S20. Representative H&E stained images of major organs collected from mice in various groups after 16 days.
